# The new 2012 German recommendations for treating rheumatoid arthritis: Differences compared to the European standpoint

**DOI:** 10.1007/s00393-012-1093-6

**Published:** 2013-02-09

**Authors:** J. Wollenhaupt, K. Albrecht, K. Krüger, U. Müller-Ladner

**Affiliations:** 1_1093 Klinik für Rheumatologie und klinische Immunologie, Schön Klinik Hamburg Eilbek, Dehnhaide 120, 22081 Hamburg, Germany; 2_1093 Deutsche Gesellschaft für Rheumatologie, Berlin, Germany; 3_1093 Praxiszentrum Rheumatologie, München, Germany; 4_1093 Abteilung für Rheumatologie und klinische Immunologie, Justus-Liebig-Universität Giessen, Kerckhoff-Klinik, Bad Nauheim, Germany

**Keywords:** Rheumatoid arthritis, Therapy, Guideline, Recommendation, Biologics, DMARDS, Rheumatoide Arthritis, Therapie, Leitlinie, Empfehlung, Biologika, Basistherapeutika

## Abstract

The German Society for Rheumatology recently published guidelines for the sequential therapy of rheumatoid arthritis (RA). These recommendations were developed as a transition from the 2010 EULAR (EUropean League Against Rheumatism) recommendations to the national clinical practice and are based on an updated systematic literature research and expert discussion. While most EULAR recommendations have remained unchanged, some were modified based on new evidence from randomized, controlled trials, current clinical practice, or national drug approval status. The guidelines also include a treatment algorithm for sequential therapy of RA with disease-modifying agents including biologics.

## Abstract (de)

Die Deutsche Gesellschaft für Rheumatologie (DGRh) hat jüngst eine S1-Leitlinie zur sequenziellen medikamentösen Therapie der rheumatoiden Arthritis veröffentlicht. Die Empfehlungen beruhen auf einer Bewertung der EULAR (EUropean League Against Rheumatism) Recommendations vor dem Hintergrund der landesüblichen klinischen Praxis auf der Basis einer aktualisierten systematischen Literaturrecherche und eines Expertenurteils. Während sich viele EULAR-Empfehlungen in der deutschen Version unverändert finden, ergaben sich für einige Änderungen auf der Grundlage neuer Evidenz aus randomisierten kontrollierten Studien, derzeitiger klinischer Praxis oder des nationalen Medikamentenzulassungsstatus. Konkretisiert werden die Empfehlungen durch einen überarbeiteten Therapiealgorithmus mit Basistherapeutika einschließlich Biologika. Der vorliegende Kommentar soll – auch für englischsprachige Leser – die Gemeinsamkeiten und Unterschiede der deutschen Empfehlungen 2012 und der EULAR Recommendations von 2010 erläutern.

On the verge of this year’s German Society of Rheumatology congress in Bochum, the new German guidelines for the medical treatment of rheumatoid arthritis have been published [1]. Based on the 2010 EULAR recommendations (EUropean League Against Rheumatism) [2] and subsequent evidence from an additional systematic literature research [3] and expert consensus, they provide not only information concerning state-of-the-art treatment of rheumatoid arthritis (RA) but also a modification of the hitherto common treatment algorithm in Germany [4].

Although the predominant part of the single EULAR recommendations remains unchanged, recent data from the current systematic literature research resulted in distinct rephrasing of the original EULAR recommendations (Tab. 1, Tab. 2). In addition, differences in the German statuary order and a somewhat different status of approval have been followed. These new guidelines have been approved by a committee of national experts and the executive committee of the German Society of Rheumatology, who have discussed and voted upon the final set of the recommendations.

**Tab. 1:** German 2012 recommendations for the medical treatment of rheumatoid arthritis

Overarching principles	
A	Rheumatologists are the specialists who should primarily care for patients with RA
B	Treatment of patients with RA should aim at the best care and is based on a shared decision between the patient and the rheumatologist
C	RA is expensive in regards to direct and indirect costs both of which should be considered by the treating rheumatologist
**12 recommendations for the medical treatment of RA**	
1	Treatment with conventional DMARDs should be started as soon as the diagnosis of RA is made
2	The target of remission or low disease activity should be reached as soon as possible. As long as the target has not been reached, adjustment of treatment and frequent monitoring is necessary
3	MTX should be the first DMARD in patients with active RA
4	When MTX cannot be used as first treatment, another conventional DMARD, e.g., leflunomide or sulfasalazine, should be considered
5	Initial combination therapy with conventional DMARDs has not demonstrated advantages to monotherapy in patients with active RA
6	Glucocorticoids should be added to the initial treatment with conventional DMARDs at low to moderately high doses
7	If the treatment target is not achieved despite optimized DMARD monotherapy, a combination therapy with conventional DMARDs should be considered. In case of high disease activity, especially in combination with poor prognostic factors, combination with a biological DMARD should be considered
8	After failure of two conventional DMARDs (in monotherapy or in combination), a biologic therapy is recommended
9	Patients with active RA, for whom a TNF inhibitor has failed as the first biologic DMARD, can switch to another TNF inhibitor, abatacept, rituximab, or tocilizumab
10	In case of refractory RA or contraindications to the previously mentioned conventional or biologic DMARDs, other DMARDs and immunmodulated therapies can be considered
11	In case of sustained remission, a stepwise tapering of the DMARD therapy should be considered as a shared decision between patient and doctor
12	Apart from disease activity, factors such as structural progression, comorbidities, safety concerns, and social aspects should be taken into account

*RA* rheumatoid arthritis, *DMARDs* disease-modifying antirheumatic drugs, *MTX* methotrexate, *TNF* tumor necrosis factor.

**Tab. 2:** The 2010 EULAR recommendations for the management of rheumatoid arthritis with nonbiological and biological disease-modifying antirheumatic drugs [2]

Overarching principles	
A	Rheumatologists are the specialists who should primarily care for patients with RA
B	Treatment of patients with RA should aim at the best care and must be based on a shared decision between the patient and the rheumatologist
C	RA is expensive in regards to medical costs and productivity costs, both of which should be considered by the treating rheumatologist
**15 recommendations for the management of RA**	
1	Treatment with synthetic DMARDs should be started as soon as the diagnosis of RA is made
2	Treatment should be aimed at reaching a target of remission or low disease activity as soon as possible in every patient; as long as the target has not been reached, treatment should be adjusted by frequent (every 1–3 months) and strict monitoring
3	MTX should be part of the first treatment strategy in patients with active RA
4	When MTX contraindications (or intolerance) are present, the following DMARDs should be considered as part of the (first) treatment strategy: leflunomide, sulfasalazine, or injectable gold
5	In DMARD naïve patients, irrespective of the addition of GCs, synthetic DMARD monotherapy rather than combination therapy of synthetic DMARDs may be applied
6	GCs added at low to moderately high doses to synthetic DMARD monotherapy (or combinations of synthetic DMARDs) provide benefit as initial short-term treatment, but should be tapered as rapidly as clinically feasible
7	If the treatment target is not achieved with the first DMARD strategy, addition of a biological DMARD should be considered when poor prognostic factors are present; in the absence of poor prognostic factors, switching to another synthetic DMARD strategy should be considered
8	In patients responding insufficiently to MTX and/or other synthetic DMARDs with or without GCs, biological DMARDs should be started; current practice would be to start a TNF inhibitor (adalimumab, certolizumab, etanercept, golimumab, infliximab) which should be combined with MTX
9	Patients with RA for whom a first TNF inhibitor has failed should receive another TNF inhibitor, abatacept, rituximab, or tocilizumab
10	In cases of refractory severe RA or contraindications to biological agents or the previously mentioned synthetic DMARDs, the following synthetic DMARD might be also considered, as monotherapy or in combination with some of the above: azathioprine, cyclosporin A (or exceptionally, cyclophosphamide)
11	Intensive medication strategies should be considered in every patient, although patients with poor prognostic factors have more to gain
12	If a patient is in persistent remission, after having tapered GCs, one can consider tapering biological DMARDs, especially if this treatment is combined with a synthetic DMARD
13	In cases of sustained long-term remission, cautious titration of synthetic DMARD dose could be considered, as a shared decision between patient and doctor
14	DMARD naïve patients with poor prognostic markers might be considered for combination therapy of MTX plus a biological agent
15	When adjusting treatment, factors apart from disease activity, such as progression of structural damage, comorbidities and safety concerns should be taken into account

*DMARD* disease-modifying antirheumatic drug, *GCs* glucocorticoids, *MTX* methotrexate, *RA* rheumatoid arthritis, *TNF* tumor necrosis factor.

The national experts agreed to leave the overarching principles and the first two EULAR recommendations without modification. They refer to the quickest-possible diagnosis and treatment of rheumatoid arthritis at its best in accordance with the patient and the rheumatologist, targeting early remission and with respect to direct and indirect costs. Furthermore, it was beyond any doubt that according to recommendation 3, methotrexate should be part of the first treatment strategy.

In case of methotrexate contraindication or intolerance, leflunomide and sulfasalazine are considered part of the first treatment strategy in both recommendations. However, injectable gold is not recommended in the current German guidelines. The main reason for not following the EULAR recommendation in this aspect and despite the available high-level evidence for the efficacy of injectable gold was the decreasing experience of the rheumatologists and the considerable side effects in long-term use.

In contrast to previous decisions, EULAR recommendation 5 took for the first time a firm stand towards a DMARD monotherapy (disease-modifying antirheumatic drug) rather than a combination therapy of synthetic DMARDs. In fact, no clinical study has demonstrated a significant advantage of combination therapy over monotherapy in the absence of glucocorticoids. Nevertheless, the EULAR recommendation also states that while in DMARD naïve patients the balance of efficacy and toxicity favors monotherapy, the respective evidence is inconclusive in DMARD inadequate responders. Here, the German guidelines only state the lacking evidence for the advantage of combination therapy and recommend a monotherapy explicitly for DMARD naïve patients. In DMARD inadequate responders, a clear recommendation remains to be determined by reason of missing evidence.

EULAR and German guidelines conform that adding glucocorticoids to DMARD monotherapy or combination therapy is beneficial for the patient. As evidence concerning doses and duration of glucocorticoid-bridging therapy is not available, both recommendations remain general and lack a more specific advice.

The EULAR recommendation 7 emphasizes the importance of prognostic factors for the further treatment decisions. A biological DMARD can be added to a synthetic DMARD if poor prognostic factors are present in DMARD inadequate responders. Otherwise, a switch to another synthetic DMARD strategy should be considered. At this point, the German guidelines strongly recommend a combination treatment of several DMARDs in DMARD inadequate responders but also allow a biological DMARD as part of the combination therapy if poor prognostic factors are present. The conclusion of both guidelines is similar, even if the order of the German statements accentuates the possibility to primarily utilize the full potential of synthetic DMARD combination.

According to the available evidence and the approval as first biological agents, the initiation of a biological therapy was mainly equalized with the initiation of a TNF (tumor necrosis factor) inhibitor in EULAR recommendation 8. This priority status of TNF inhibitors has been withdrawn in the German guidelines as the more recent biologic DMARDs abatacept and tocilizumab provided equivalent evidence for their efficacy and safety and are also approved as first-line biological agents. As indirect treatment comparison show similar efficacy for all biologic agents except for anakinra, no specific agents are recommended at this point for preferable first line therapy.

The switch to a second biologic treatment after an inadequate response to the first biological therapy remains identical in the EULAR and German guidelines—as second TNF inhibitor, abatacept, rituximab or tocilizumab are possible agents without a specific ranking. However, full efficacy and safety data for a change to a defined second biologic agent following abatacept or tocilizumab are still lacking.

In the case of rheumatoid arthritis refractory to several DMARDs and biologic agents, azathioprine, cyclosporin A and cyclophosphamide are specifically recommended by EULAR due to existing evidence on their efficacy, of course with respect to their individual toxicity. However, German recommendation 10 has been rephrased to a more general statement in order to also enable the application of other treatment options with the necessary reduction of evidence level to expert opinion.

The final recommendations conform to the EULAR statements, even if recommendations 12 and 13 are combined in the German guidelines—specific suggestions concerning the procedure in case of a sustained remission are not provided due to the lack of evidence. Intensive treatment strategies and treatment adjustment considering structural progression, comorbidities and safety concerns are self-evident. EULAR recommendation 14 refers to DMARD naïve patients again and creates the possibility to begin a biologic agent in combination with MTX as a first treatment strategy in individual patients with poor prognostic factors. By reason of order, this exceptional case was included and discussed in recommendation 3 of the German guidelines.

The aligned treatment algorithm summarizes the recommendations and represents the current practice subjected to the different strategy steps in the course of the disease (Fig. 1).

**Fig. 1:**
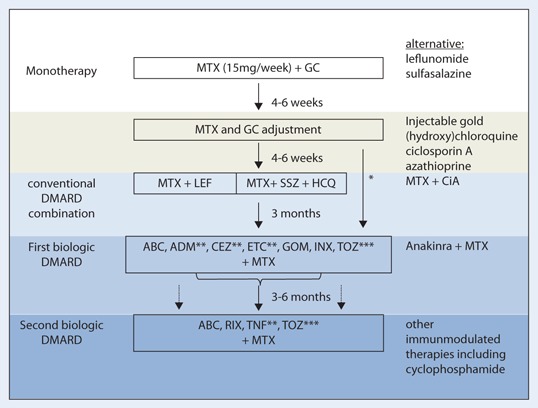
Algorithm based on the German 2012 recommendations for the medical treatment of rheumatoid arthritis. *ABC* abatacept, *ADM* adalimumab, *CEZ* certolizumab, *ETC* etanercept, *GOM* golimumab, *INX* infliximab, *RIX* rituximab, *TOZ* tocilizumab, *GC* glucocorticoids, *CiA* ciclosporin A, *HCQ* hydroxychloroquine, *LEF* leflunomide, *MTX* methotrexate, *SSZ* sulfasalazine, *TNF* TNF inhibitors. *high disease activity, especially in combination with poor prognostic factors, **in case of MTX contraindication, ADM, CTZ, ETC are also approved in monotherapy, ***in case of MTX contraindication, TOZ is also approved in monotherapy and has demonstrated similar efficacy in monotherapy as well as in combination with MTX

Taken together, the new 2012 German recommendations provide an update of the current evidence for the medical treatment on rheumatoid arthritis on the basis of the 2010 EULAR recommendations, providing an evidence-based real-life set of recommendations for the use in the daily practice of every rheumatologist in (and outside) of Germany.
